# Plant Hormones Differentially Control the Sub-Cellular Localization of Plasma Membrane Microdomains during the Early Stage of Soybean Nodulation

**DOI:** 10.3390/genes10121012

**Published:** 2019-12-05

**Authors:** Zhenzhen Qiao, Prince Zogli, Marc Libault

**Affiliations:** 1 Department of Microbiology and Plant Biology, University of Oklahoma, Norman, OK 73019, USA; qiaoz@ornl.gov; 2 Biosciences Division, Oak Ridge National Laboratory, Oak Ridge, TN 37830, USA; 3 Department of Agronomy and Horticulture, University of Nebraska-Lincoln, Beadle Center, Lincoln, NE 68503, USA; pzogli2@unl.edu

**Keywords:** root hair, legume, nodulation, plasma membrane microdomains, phytohormones

## Abstract

Phytohormones regulate the mutualistic symbiotic interaction between legumes and rhizobia, nitrogen-fixing soil bacteria, notably by controlling the formation of the infection thread in the root hair (RH). At the cellular level, the formation of the infection thread is promoted by the translocation of plasma membrane microdomains at the tip of the RH. We hypothesize that phytohormones regulate the translocation of plasma membrane microdomains to regulate infection thread formation. Accordingly, we treated with hormone and hormone inhibitors transgenic soybean roots expressing fusions between the Green Fluorescent Protein (GFP) and *Gm*FWL1 or *Gm*FLOT2/4, two microdomain-associated proteins translocated at the tip of the soybean RH in response to rhizobia. Auxin and cytokinin treatments are sufficient to trigger or inhibit the translocation of *Gm*FWL1 and *Gm*FLOT2/4 to the RH tip independently of the presence of rhizobia, respectively. Unexpectedly, the application of salicylic acid, a phytohormone regulating the plant defense system, also promotes the translocation of *Gm*FWL1 and *Gm*FLOT2/4 to the RH tip regardless of the presence of rhizobia. These results suggest that phytohormones are playing a central role in controlling the early stages of rhizobia infection by regulating the translocation of plasma membrane microdomains. They also support the concept of crosstalk of phytohormones to control nodulation.

## 1. Introduction

Nodulation is a mutualistic symbiotic interaction between the root system of plants (most commonly legumes) and rhizobia. This symbiosis leads to the development of a new root organ, the nodule, where differentiated bacteria fix and assimilate the atmospheric dinitrogen for the plant [1,2,3,4]. This highly specific symbiotic relationship is initiated with the exchange of molecular signals between the plant and rhizobia: plant flavonoids and iso-flavonoids are first recognized by the bacteria (*Bradyrhizobium diazoefficiens* in the case of the soybean plant) leading to the induction of the expression of the bacterial nodulation genes [5]. These genes are required for the synthesis and secretion of lipochito-oligosaccharides known as Nod factors (NFs) [6]. NFs are perceived by plant receptor lysine kinases localized in the plasma membrane of the root hair cell (RH; e.g., the Medicago RECEPTOR-LIKE KINASE3 (LYK3) [7]). The transfer of bacterial effector proteins into the plant cell via the type-III and type-IV secretion systems is another critical molecular mechanisms used to promote the nodulation process [8,9,10,11].

The recognition between the host plant and its symbiont will trigger molecular, cellular, and physiological responses of the plant RHs [2,12]. For instance, forward and reverse genetic studies revealed the role of plant genes in controlling the perception and subsequent infection of the plant RHs by rhizobia, notably leading to the characterization of the symbiosis signaling pathway gene network [3]. This pathway controls the curling of the RH to trap rhizobia into an infection pocket and the activation of a transcriptional response of the RHs including the transient activation of the plant defense system [13,14]. These changes will ultimately lead to the formation of the infection thread, a tubular structure that rhizobia use as a tunnel to infect dividing root cortical cells, thus forming nodule primordia then nodules. Later the bacteria differentiated into bacteroids will infect the dividing cortex cells of the nodule.

Both infection thread formation and cortex cell infection are considered as endocytosis-like processes [15]. In plants, two independent endocytosis mechanisms exist: clathrin-mediated endocytosis and membrane microdomain-mediated endocytosis, which is initiated by the internalization of clusters of membrane microdomains, subdomains of the plasma membrane characterized by their high content in sphingolipids and specific protein composition [16,17,18]. The former was notably highlighted upon characterization of the role of the clathrin heavy chain1 (CHC1) protein as a positive regulator of *Lotus japonicus* nodulation [19]. Regarding the latter, several studies revealed the role of microdomains in triggering the formation of the infection thread and, later, the infection of nodule cells by bacteroids. For instance, the medicago flotillins 2 and 4 (FLOT2 and FLOT4) and the soybean FW2.2-like 1 (FWL1) microdomain-associated proteins, are quickly translocated to the RH tip in response to rhizobia inoculation to promote RH infection [20,21,22,23,24]. In addition, GmFWL1 has been observed in the symbiosome membrane while MtFLOT4 localized in the membrane of the infection thread only. The medicago receptor Lysine Kinase 3 (LYK3) also participates to rhizobia infection upon its recruitment in MtFLOT4-SYMREM1-labeled microdomains [25]. The SYMREM1 remorin protein, another microdomain-associated protein localized in the infection thread and symbiosome plasma membrane, is proposed to act as a scaffolding protein to control rhizobia endocytosis and its release into the host cytoplasm [26,27]. The role of microdomain-associated proteins in promoting the microbial infection of plant cell is not restricted to mutualistic symbiotic microbes but also to pathogenic microbes such as the infection of tomato plants by the Potato virus X [28]. To date, the molecular and physiological mechanisms regulating the translocation of microdomains in the RH plasma membrane in response to rhizobia remain unknown.

Plant hormones play important roles in controlling both nodulation and endocytosis processes [29,30,31,32]. For instance, cytokinin controls the formation of the nodule primordia and is a major player of the autoregulation of legume nodulation notably by inhibiting root hair cell infection and infection thread formation [33,34,35,36]. The induction of the formation of nodule primordia by cytokinin is the consequence of the activation of the expression of the nodule inception (*NIN*) genes in the pericycle. These genes encode RWP-RK transcription factors responsible for the activation of cortical cell divisions, the initiation of a nodule meristem, and ultimately, nodule development [37,38,39,40]. The induction of nodule formation by cytokinin is also supported by the development of nodule primordia of non-nodulation mutants such as *nfr1*, *nfr5*, *symRK*, *nup133*, *nup85*, *castor*, *pollux*, and *ccamk*, upon cytokinin treatment [38].

In response to nod factor treatment, auxin concentration and the expression of the auxin-responsive gene *GH3* increase in root hair in *L. japonicus* [41] and medicago [42], suggesting that the accumulation of auxin in legume root hairs is a conserved mechanism regulating the early stages of the rhizobia symbiosis pathway. The polar transport of auxin is also critical in the formation of nodules [43,44,45]. Changes induced in auxin transport by the use of chemical drugs (e.g., (1-naphthyl) phthalamic acid and 2,3,5-triiodobenzoic acid (TIBA)) are sufficient to induce the formation of pseudo-nodules in medicago [46,47]. The active and directional transport of auxin across the plasma membrane is accomplished through transmembrane localized auxin transporters such as the PINFORMED (PIN) proteins [48,49,50,51,52]. The inhibition of auxin transport results in the initiation of the formation of indeterminate nodules (e.g., medicago [53] and *Vicia sativa* [54]) but not in the initiation of determinate nodules [55]. Altering auxin signaling also affects nodule formation. For instance, upon overexpression of the flavin monooxygenase Gm*YUC2a* gene, auxin accumulates in the root leading to a delay in nodule formation and a decrease in nodule number [56]. Oppositely, the silencing of the auxin response factor GmARF8a/b via miR167 positively regulates nodule number in soybean [57] while the overexpression of miR160 enhances auxin responsiveness and reduces the nodule number in medicago [58] and soybean [43,59]. Auxin also plays a critical role in controlling the development of medicago indeterminate nodules as well as lotus and soybean determinate nodules [43,60,61]. Sanko-Sawczenko et al. recently described the central role of polar auxin transport and auxin homeostasis in controlling determinate nodule development and differentiation [62].

Increasing the complexity of the hormonal crosstalk, the role of salicylic acid (SA) varies depending nodule determination. For instance, SA repressed the development of indeterminate nodules but seems to have no effect on the development of determinate nodules [63]. SA has also opposite effects on the formation of infection threads and the development of nodules depending on its concentration and across legume species [64,65].

To finely regulate the nodulation process, auxin and CK are involved in a regulatory feedback loop [66,67]. The balance between auxin and CK concentration and its role in controlling nodule development was recently highlighted by using the *DR5* auxin- and *TCSn* CK-inducible synthetic promoters fused to fluorescent proteins [68]. To regulate the direction of auxin fluxes, cytokinin regulates the distribution of PIN1, which is strongly associated with plasma membrane microdomains [69]. Interestingly, the translocation of the PIN proteins is not only regulated by CK but by other phytohormones such as GA and auxin itself [48,70]. SA also affects PIN protein trafficking by inhibiting bulk transport dependent on clathrin-mediated endocytosis [48]. A recent report showed the exogenous SA application activates auxin synthesis and regulates auxin transport in a dose dependent manner. This accumulation of auxin increases the number of root cell layers [71].

Altogether, these studies highlight the crosstalk, transport, and local accumulation of phytohormones at the site of rhizobia infection. These events are required to control the nodulation process and the signaling events occurring at the level of the plasma membrane [32]. Furthermore, hormone pathway crosstalk is the result of antagonist roles of various plant hormones in controlling the formation of the infection thread and the development of nodules [12,72]. In this study, we provide evidence of the role of phytohormones in positively or negatively controlling the distribution of root hair plasma membrane microdomains during nodulation. More specifically, using *Gm*FWL1 and *Gm*FLOT2/4 as markers of plasma membrane microdomains, we demonstrated that, auxin, cytokinin, and SA differentially regulate the translocation of microdomains in the RHs independently of the inoculation of the plant by *B. diazoefficiens*.

## 2. Materials and Methods

### 2.1. Bacterial Culture

*Escherichia coli* DH5α and *Agrobacterium rhizogenes* K599 strains were grown overnight in Luria–Bertani medium supplemented with appropriate antibiotics at 37 °C and 30 °C, respectively. *B. diazoefficiens* USDA110 was grown at 30 °C for 3 days in HM medium [73] supplemented with 0.025% yeast extract, 0.1% D-Arabinose, and 0.004% chloramphenicol. Upon hairy root transformation (see below), *B. diazoefficiens* cells were pelleted (4000 rpm for 10 min), washed, and resuspended with sterile water to OD_600nm_ = 0.3 before to inoculate soybean transgenic roots.

### 2.2. Plasmids Construction

The *pCvMV::GFP, pCvMV::GmFWL1-GFP, pCvMV::mCherry*, and *pCvMV::mCherry-GmFLOT2/4* were all generated using the multisite Gateway system (Invitrogen, Carlsbad, CA, USA) as previously described [23]. *A. rhizogenes* (strain K599) was transformed with the four plasmids in order to generate transgenic soybean root system using the hairy root transformation protocol.

### 2.3. Plant Transformation

*Glycine max* L. (Merrill) Williams 82 plants were used for plant transformation as previously described [74]. Briefly, overnight liquid cultures of the *A. rhizogenes* K599 strains carrying the transgenes of interest were centrifuged at 4000 rpm for 10 min at room temperature and resuspended in water supplemented with 20 µM acetosyringone to OD_600nm_ = 0.3–0.35. Five mL of the *A. rhizogenes* suspension were inoculated onto a sterilized piece of rockwool placed on a clean petri dish. The soybean shoot located above the first true leaf of two weeks-old plants was cut off and inserted into each piece of rockwool inoculated with *A. rhizogenes*. Three days after transformation, the shoot explants were watered with nutritive N-PNS solution and allowed to grow for additional 10 days. On the tenth day, the plants were transferred from the rockwool into the ultrasound aeroponic system [75] or autoclaved vermiculite perlite mixed at 3:1 ratio and grown for 3–4 weeks to allow the development of the plant root system.

### 2.4. Plant Treatments

To validate the treatment of soybean roots with phytohormones (i.e., auxin) and auxin inhibitors, soybean composite plants transformed with the *DR5::GUS* auxin inducible and the *DR5-REV::GUS* transgenes were incubated for 6 days in nitrogen-free B&D solution supplemented with L-kynurenine (1.5 μM) [76] and 2,3,5-triiodobenzoic acid (TIBA, 10 μM) [77]. Upon treatment, plants were incubated for 24 h into fresh nitrogen-free B&D solution supplemented with auxin inhibitors. As a control, *DR5::GUS* plants were kept in nutritive solution without inhibitors or with auxin treatment. Upon treatment and GUS staining, the transgenic roots were observed using a Leica MZ10 Stereo microscope

To observe the role phytohormones on the translocation of plasma membrane-associated microdomains, four weeks-old composite soybean root system were treated with the mist of nitrogen-free B&D solution [78] supplemented with 1 μM 2,4-dichlorophenoxyacetic acid (2,4-D) [52], 100 μM of salicylic acid (SA) [63], or 1 μM 6-Benzylaminopurine (6-BA) [36]. Phytohomone concentrations were selected based on previous studies as noted above. Due to the instability of natural IAA [52], we used the synthetic 2,4-D for auxin treatment. Control plants were treated with a mist of the nitrogen-free B&D solution. One day after hormone treatment, suspension of *B. diazoefficiens,* USDA110 (OD_600nm_ = 0.1) was sprayed on the root. The transgenic *pCvMV::GFP, pCvMV::GmFWL1-GFP, pCvMV::mCherry* and *pCvMV::mCherry*-*GmFLOT2/4* roots were observed one day after rhizobia inoculation, using an Olympus FluoView 500 laser scanning confocal microscope with an argon 488 nm laser line and a helium-neon 543 nm laser line, respectively.

To test the effect of auxin biosynthesis and transport inhibitors on the translocation of plasma-membrane microdomains in soybean RHs, four weeks-old composite soybean root system were pre-incubated for 6 days in nitrogen-free B&D solution supplemented with L-kynurenine (1.5 μM) and 2,3,5-triiodobenzoic acid (10 μM). Upon treatment, plants were then moved into fresh nitrogen-free B&D solution supplemented with inhibitors and incubated for 24 h before imaging using a Nikon A1 confocal microscope. To infer effect of auxin inhibitors on GmFWL1 translocation, inhibitor treated plants were inoculated with *B. diazoefficiens*.

The microscopic observations of the subcellular localization of microdomain-associated proteins were conducted on three independent replicates of transgenic soybean. For each replicates and conditions, three to five individual composite soybean plants were generated. The transgenic roots expressing the GFP/mCherry were selected under an epi-fluorescent stereomicroscope. Then, the transgenic root hair cells located in the root elongation zone were observed under a confocal microscope.

## 3. Results

### 3.1. Testing and Validating the Treatment of Composite Soybean Plants with Phytohormones and Plant Hormone Inhibitors

The ultrasound aeroponic system has been utilized as an effective plant growth system to observe the development of plant roots and to induce the formation of high density of RHs on the primary and lateral roots. The ultrasound aeroponic system has also been used to inoculate legumes with rhizobia to induce nodulation, and to generate and grow composite plants [75]. Having the objective to analyze the role of phytohormones in controlling the translocation of RH plasma membrane microdomains in response to rhizobia, we tested the possibility to homogeneously treat soybean composite plants with phytohormones and hormone inhibitors.

The synthetic auxin-inducible *DR5* promoter has been broadly used across different plant species, including soybean, to monitor auxin concentration in the root system [43,79,80]. Hence, using the *DR5:GUS* reporter gene encoding the β-glucuronidase, we treated soybean *DR5::GUS* composite plants with a plant nutritive solution supplemented with auxin (i.e., 2,4-dichlorophenoxyacetic acid). As a control, we also generated composite plants transformed with the *DR5* promoter but cloned in the reverse orientation (*DR5-REV*) upstream to the *GUS* reporter gene.

**Figure 1:**
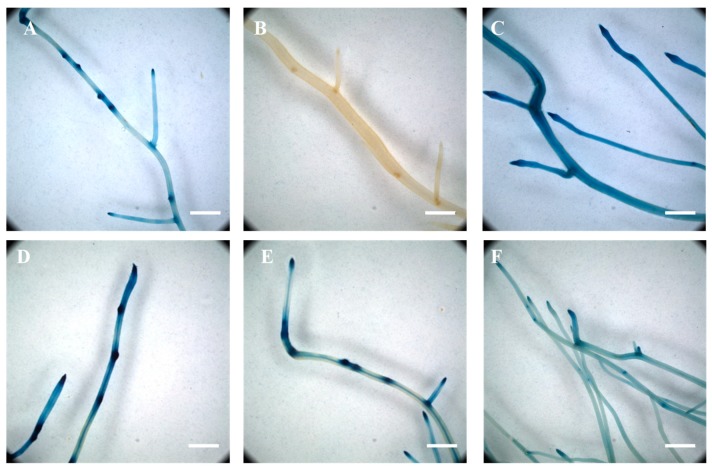
Transcriptional activity of the auxin-inducible *DR5* synthetic promoter in response to 2,4-dichlorophenoxyacetic acid and auxin inhibitor treatments. The endogenous concentration of auxin was revealed in soybean transgenic roots expressing the *DR5::GUS* reporter (**A**). The *DR5-REV::GUS* transgenic roots did not show any β-glucuronidase activity (**B**). Upon auxin treatment, the *DR5* promoter was ubiquitously active in the soybean transgenic roots (**C**). While individual treatment of the plants with L-kynurenine, an inhibitor of auxin biosynthesis (**D**), and TIBA, an inhibitor of auxin transport (**E**), did not significantly affect the activity of the *DR5* synthetic promoter, a combined treatment of the two drugs was sufficient to reduce *DR5::GUS* activity in the entire soybean root system (**F**). Scale = 200 µm.

As previously published [43,79], under control conditions (i.e., no phytohormone supplemented), β-glucuronidase activity was mostly limited to the location of the emergence of lateral roots (Figure 1A). As expected, the soybean *DR5-REV* composite plants did not show any β-glucuronidase activity (Figure 1B). Upon auxin treatment, the activity of the *DR5* promoter was ubiquitous in the root system (Figure 1C). We observed a similar result when plants were grown and treated with auxin in the ultrasound aeroponic system. This result supports the effective treatment of plant roots with auxin.

To further validate our methodology to treat composite plants with chemical compounds, we also supplemented the plant nutritive solution with L-kynurenine, an inhibitor of auxin biosynthesis, and 2,3,5-triiodobenzoic acid, an inhibitor of auxin transport [76,77]. A preliminary assay did not reveal a decrease in *DR5::GUS* activity after one day of auxin inhibitors. Six days of treatment of the soybean plant with L-kynurenine or 2,3,5-triiodobenzoic acid alone did not result in a noticeable change in the activity of the *DR5::GUS* reporter gene (Figure 1D,E) compared to untreated plants (Figure 1A). However, the concomitant use of the two drugs during 6 days of treatment led to a decrease and more diffuse activity of the *DR5* promoter (Figure 1F) compared to untreated *DR5::GUS* composite plants (Figure 1A). Based on our results, the soybean root system can be homogeneously treated with various chemical compounds including, for the purpose of this study, phytohormones and auxin inhibitors. In addition, our results validate the use of a cocktail of auxin inhibitors to modify the accumulation of auxin into the soybean root system.

### 3.2. Auxin Induces the Translocation of Plasma Membrane-Associated Microdomains at the Tip of the Soybean RHs

Several phytohormones control plant-microbe interactions, legume nodulation, and plant endocytosis processes [29,31,65,81]. These biological processes are also regulated by the translocation of plasma membrane microdomains. Accordingly, we hypothesized that plant hormones might control the translocation of plasma membrane microdomains in order to regulate the nodulation process including the invagination of the plasma membrane to initiate the formation and progression of the infection thread, then the infection of plant nodule cells by bacteroids.

**Figure 2:**
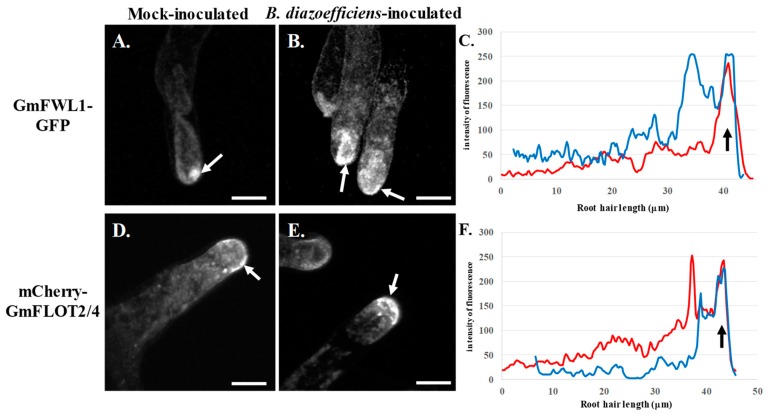
Subcellular localization of GmFWL1-GFP (**A**–**C**) and mCherry-GmFLOT2/4 (**D**–**E**) in mock- (**A**,**D**) and *B. diazoefficiens*-inoculated soybean root hairs (**B**,**E**) in response to 2,4-dichlorophenoxyacetic acid treatment. Soybean root hair cells expressing the GmFWL1-GFP and mCherry-GmFLOT2/4 fusion proteins were treated with 2,4D then inoculated or not with *B. diazoefficiens*. White arrows highlight the accumulation of chimeric proteins at the tip of the root hair cells upon auxin treatment. C and F: Quantification of the intensity of the fluorescence (y-axis) of the GmFWL1-GFP (**C**) and mCherry-GmFLOT2/4 proteins (**F**) in mock- (red) and *B. diazoefficiens*-inoculated (blue) and 2,4-dichlorophenoxyacetic-treated soybean root hair cells (x-axis, µm; the black arrow highlight the position of the root hair tip). Scale = 100µm. The pictures shown are representative from a series of observations conducted on three independent biological experiments.

To verify our hypothesis, we looked for changes in the sub-cellular localization of two soybean microdomain-associated proteins, GmFWL1 and GmFLOT2/4, in response to phytohormone treatments. These two proteins were selected for this study based on their previous characterization as microdomain-associated proteins, and their characteristic translocation at the tip of the soybean RH in response to rhizobia inoculation [20,23]. Accordingly, we expressed *Gm*FWL1-GFP and mCherry-GmFLOT2/4 chimeric proteins in the root system of soybean composite plants growing in the ultrasound aeroponic system. Auxin was supplemented to the plant nutritive solution and applied on transgenic composite root systems. Upon treatment, the transgenic plants were inoculated or not with *B. diazoefficiens*. Looking for changes in the spatial distribution of the two microdomain-reporter proteins, both *Gm*FWL1-GFP and mCherry-*Gm*FLOT2/4 chimeric proteins were translocated at the tip of the RH as soon as one day after auxin treatment and independently of *B. diazoefficiens* inoculation (Figure 2). The pattern of localization of the microdomains and the timing of their translocation at the tip of the root hair (i.e., as early as 24 h after treatment; Figure 2C,F; Supplemental Figure S1) were similar to those previously reported in response to *B. diazoefficiens* inoculation [23]. Our observations suggest that the exogenous treatment of soybean plants by auxin was sufficient to induce the translocation of microdomains at the tip of the soybean root hair cell, one of the early cellular responses of the soybean RH to rhizobia. This cellular function complemented the molecular role of auxin in inducing auxin-responsive genes in the RH upon rhizobia inoculation [14].

### 3.3. Inhibition of Auxin Fluxes Reduced the Translocation of GmFWL1 to RH Tip

Local accumulation of auxin through the regulation of auxin fluxes by auxin transporters (e.g., PIN proteins) is required to trigger legume nodulation. To provide further evidence for the role of auxin fluxes in controlling the redistribution of plasma membrane-associated microdomains at the tip of the RHs in response to rhizobium, we treated *B. diazoefficiens*-inoculated and mock-inoculated *Gm*FWL1-GFP transgenic soybean roots with L-kynurenine alone, an inhibitor of auxin biosynthesis and with a cocktail of L-kynurenine and 2,3,5-triiodobenzoic acid, an inhibitor of auxin transport as previously described (Figure 1).

**Figure 3:**
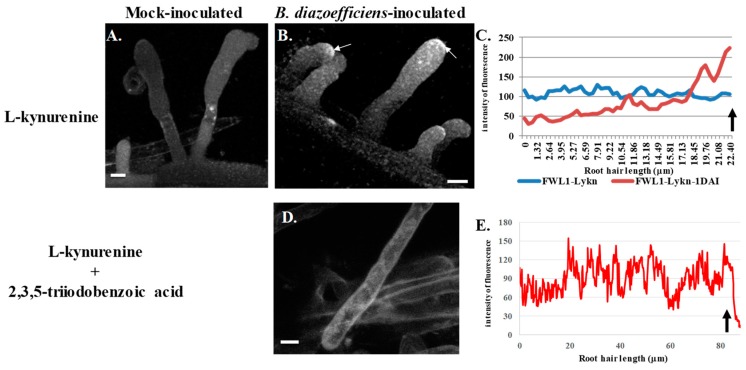
Subcellular localization of GmFWL1-GFP in soybean root hairs after treatment with L-kynurenine, an auxin biosynthesis inhibitor (**A**–**C**), or after a treatment with L-kynurenine and 2,3,5-triiodobenzoic acid, an inhibitor of auxin transport (**D**,**E**). Twenty-four hours after treatment, the plants were mock-inoculated (**A**) or inoculated with *B. diazoefficiens* (**B**,**D**). Soybean root hair cells expressing the GmFWL1-GFP chimeric protein were observed 24 h after inoculation under an epifluorescent confocal microscope. The chimeric protein accumulates at the tip of the root hair in response to *B. diazoefficiens* only upon L-kynurenine treatment (white arrows). (**C**,**E**): Quantification of the intensity of the GmFWL1-GFP signal (y-axis) across transgenic soybean root hair cells (x-axis, μm) in inoculated (Lykn-1DAI) and mock-inoculated L-kynurenine-treated (Lykn) transgenic root hair cells (**C**), and in inoculated L-kynurenine/2,3,5-triiodobenzoic acid transgenic root hair cells (**E**; the arrow highlight the position of the root hair tip). Scale = 10 μm. The pictures shown are representative from a series of observations conducted on three independent biological experiments.

Treatment with L-kynurenine alone, which is not sufficient to affect auxin accumulation in the soybean root (Figure 1D), did not affect the translocation of the GmFWL1 protein in response to rhizobia (Figure 3A–C; Supplemental Figure S1). However, upon concomitant treatments with L-kynurenine and TIBA, which were sufficient to alter auxin accumulation in the root (Figure 1F), *Gm*FWL1 did not accumulate at the tip of the RH upon *B. diazoefficiens* inoculation (Figure 3D,E; Supplemental Figure S1), similar to its localization in mock inoculated RHs. These results suggest that the perturbation of auxin transport and auxin biosynthesis was needed to inhibit the translocation of plasma membrane microdomains at the tip of the RH in response to rhizobia inoculation. These results were consistent with the notion that auxin plays a critical role during the early stages of the nodulation process [43,44,45].

### 3.4. Cytokinin and Salicylic Acid Regulate the Translocation of Plasma Membrane-Associated Microdomains at the Tip of the Soybean RHs

The transport of auxin is under the control of various phytohormones including cytokinin and salicylic acid. We hypothesized that these hormones also influence the translocation of root hair microdomains in response to rhizobia by controlling auxin fluxes. Accordingly, we treated transgenic soybean roots expressing the *Gm*FWL1-GFP or mCherry-*Gm*FLOT2/4 with cytokinin and salicylic acid then inoculated these plants with *B. diazoefficiens*.

**Figure 4:**
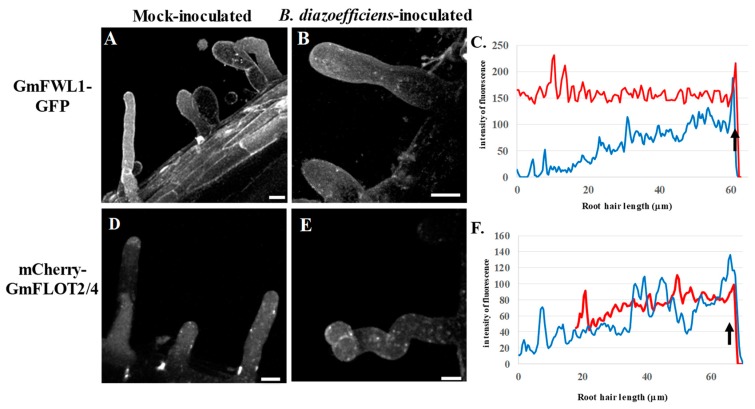
Subcellular localization of GmFWL1-GFP (**A**–**C**) and mCherry-GmFLOT2/4 (**D**,**E**) in mock- and *B. diazoefficiens*-inoculated soybean root hairs in response to cytokinin treatment. C and F: Quantification of the intensity of the fluorescence (y-axis) of the GmFWL1-GFP (**C**) and mCherry-GmFLOT2/4 proteins (**F**) in mock- (red) and *B. diazoefficiens*-inoculated (blue) and cytokinin-treated soybean root hair cells (x-axis, μm; the black arrow highlight the position of the root hair tip). Scale = 10 μm. The pictures shown are representative from a series of observations conducted on three independent biological experiments.

Upon cytokinin treatment, we did not observe significant changes in the subcellular localization of either *Gm*FWL1-GFP (Figure 4C; Supplemental Figure S1) or mCherry-*Gm*FLOT2/4 (Figure 4F; Supplemental Figure S1) in the presence or absence of *B. diazoefficiens*. This result suggests that, oppositely to auxin, cytokinin does not promote the translocation of plasma microdomains to the tip of the root hair and, as a consequence, the initiation of the formation of the infection thread. In addition, cytokinin treatment also inhibited the translocation at the tip of the root hair cells of *Gm*FWL1 and *Gm*FLOT2/4 in response to *B. diazoefficiens*. Our observations highlighted the suppression of the translocation of microdomain at the tip of the root hair cell as one cellular mechanism explaining the inhibitory effect of cytokinin on the initiation of new rhizobia infection by nitrogen-fixing symbiotic bacteria.

**Figure 5:**
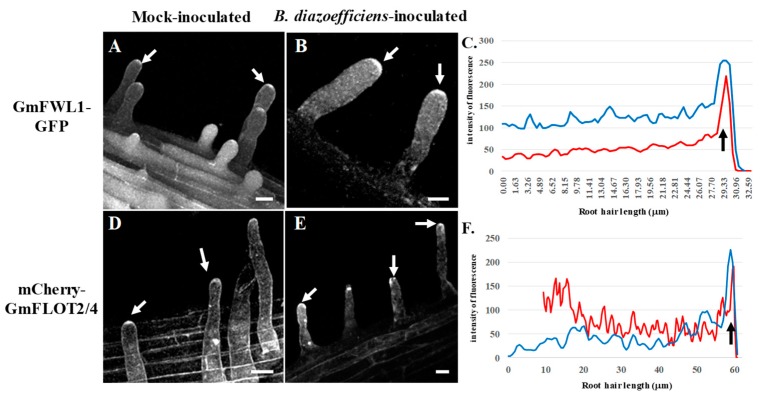
Subcellular localization of GmFWL1-GFP (**A**–**C**) and mCherry-GmFLOT2/4 (**D**,**E**) in mock- and *B. diazoefficiens*-inoculated soybean root hairs in response to salicylic acid treatment. White arrows highlight the accumulation of chimeric proteins at the tip of the root hair cells upon auxin treatment. C and F: Quantification of the intensity of the fluorescence (y-axis) of the GmFWL1-GFP (**C**) and mCherry-GmFLOT2/4 proteins (**F**) in mock- (red) and *B. diazoefficiens*-inoculated (blue) and salicylic acid-treated soybean root hair cells (x-axis, μm; the black arrow highlight the position of the root hair tip). Scale = 10 μm. The pictures shown are representative from a series of observations conducted on three independent biological experiments.

Surprisingly, salicylic acid treatments were sufficient to lead to the accumulation of *Gm*FWL1 and *Gm*FLOT2/4 at the tip of the RH independently to *B. diazoefficiens* inoculation (Figure 5; Supplemental Figure S1). The similarity existing between salicylic acid and auxin treatments on the translocation of root hair microdomains raises the question of the role of salicylic acid in regulating the early events of determinate nodulation and its interplay with auxin.

## 4. Discussion

Plasma membrane-associated proteins (e.g., flotillin, remorin, and FWL1 proteins) are important regulators of legume nodulation. Notably, the translocation of these proteins at the tip of the root hair cells in response to rhizobia is necessary to the formation then elongation of the infection thread. This biological process is considered as an endocytosis-like process [15]. This statement is supported by the identification of plasma membrane intrinsic proteins and vacuolar ATPases [24], proteins implicated in endocytosis, as microdomain-associated proteins and *Gm*FWL1 interacting partners.

Phytohormones also regulate the endocytic process notably to control plant-microbe interactions [31,43,44,52,82,83]. In this study, we provided evidence of the promoting or inhibiting role of plant hormones in controlling the translocation of root hair microdomains in order to regulate the nodulation process. More specifically, we showed that auxin fluxes induced the early responses of the soybean RHs to *B. diazoefficiens* inoculation by controlling the accumulation of GmFWL1 and GmFLOT2/4 proteins at the root hair tip.

**Figure 6:**
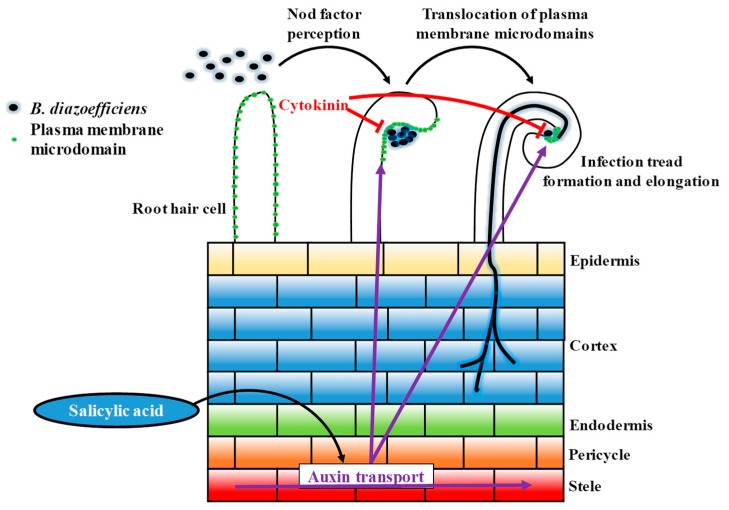
Schematic model of the regulation of auxin and cytokinin during early rhizobia infection event. Plant auxin is transported towards the root tip, also transported in acropetal direction (from root tip to elongation zone). After rhizobia infection, acropetal auxin transport at the inner cortical, endodermal and/or pericycle. The reduction of acropetal auxin transport increases auxin concentration at the rhizobia infection site, the location of a future nodule primordium, to promote nodule development. After rhizobia infection, cytokinin biosynthesis is up-regulated and perceived at the inner cortex. The increased level of cytokinin can inhibit the infection thread progression.

While cytokinins promote the formation of nodule primordia, they also inhibit root hair cells infection to autoregulate nodulation [30]. In this study, the treatment of the soybean root system with cytokinins was sufficient to prevent the typical translocation of the root hair plasma membrane microdomains at the tip of the root hair cell in response to rhizobia inoculation. This result supports the inhibitory effect of cytokinins on the early stages of the nodulation process, and provides evidence of the role of cytokinins on the biology of the root hair cell. Taken together, our data highlights the opposite role of auxin and cytokinin in controlling root hair cell infection. These data support the regulatory feedback loop existing between these two hormones to regulate legume nodulation. Based on our observation, this loop notably regulates the translocation of the microdomain protein in the RH, presumably to tightly control the initial infection process of the root hair cell by rhizobia (Figure 6).

Previous reports suggested that the salicylic acid acts as a negative regulator or a no-regulator of legume nodulation depending nodule determination [63,64,65]. Intriguingly, we observed that SA treatment was sufficient to induce the translocation of plasma membrane microdomains at the tip of the soybean root hair independently to *B. diazoefficiens* inoculation. This observation coupled with earlier scientific publications support the notion that a transient and controlled activation of the plant defense is required for a successful infection of the RH by rhizobia [13,14]. This conclusion is supported by previous studies showing that, when applied at specific concentrations, salicylic acid also promotes nodulation [63,64,65]. Our observations also suggest that both auxin and salicylic acid synergistically act together or positively influence each other to promote the early stages of soybean nodulation. Here, we proposed a model where salicylic acid positively acted on auxin fluxes to promote the infection of soybean root hair cells by rhizobia (Figure 6). This statement is supported by a recent report from Pasternak et al., (2019) who demonstrated that the exogenous application of salicylic acid was sufficient to regulate the activity of the auxin-inducible *DR5* promoter [71].

Our work highlighted the role of plant hormones in controlling an important cellular process in the soybean root hair cells: the translocation of plasma membrane-associated microdomains at the tip of the root hair cell to initiate rhizobia infection. Our work also clearly highlighted the opposite effect of auxin, salicylic acid, and cytokinin in controlling this translocation; a new role for these phytohormones as part of the regulatory feedback loop of legume nodulation.

## Supplementary Materials

The following are available online at https://www.mdpi.com/2073-4425/10/12/1012/s1, Figure S1: Quantification of the intensity of the fluorescence (y-axis) of the GmFWL1-GFP (A–D, H) and mCherry-GmFLOT2/4 proteins (E, F and G) in mock- (Red) and *B. diazoefficiens*-inoculated (Blue) soybean root hair cells (x-axis, μm), under treatments of Auxin (A and E), Cytokinin (B and F), SA (C and G), and in response to L-kynurenine (D) or L-kynurenine and 2,3,5-triiodobenzoic acid (H). The figure showed average intensity of 3 root hair cells, and the error bar showing the standard error of the intensity of the fluorescence of the GFP and mCherry proteins.
