# Supplementary material for: Plant Hormones Differentially Control the Sub-Cellular Localization of Plasma Membrane Microdomains during the Early Stage of Soybean Nodulation
Source: Genes (Basel). 2019 Dec 5;10(12):1012. doi: 10.3390/genes10121012 (PMC6947267; doi:10.3390/genes10121012)
Supplement: Supplementary file 1 [file genes-10-01012-s001.pdf]

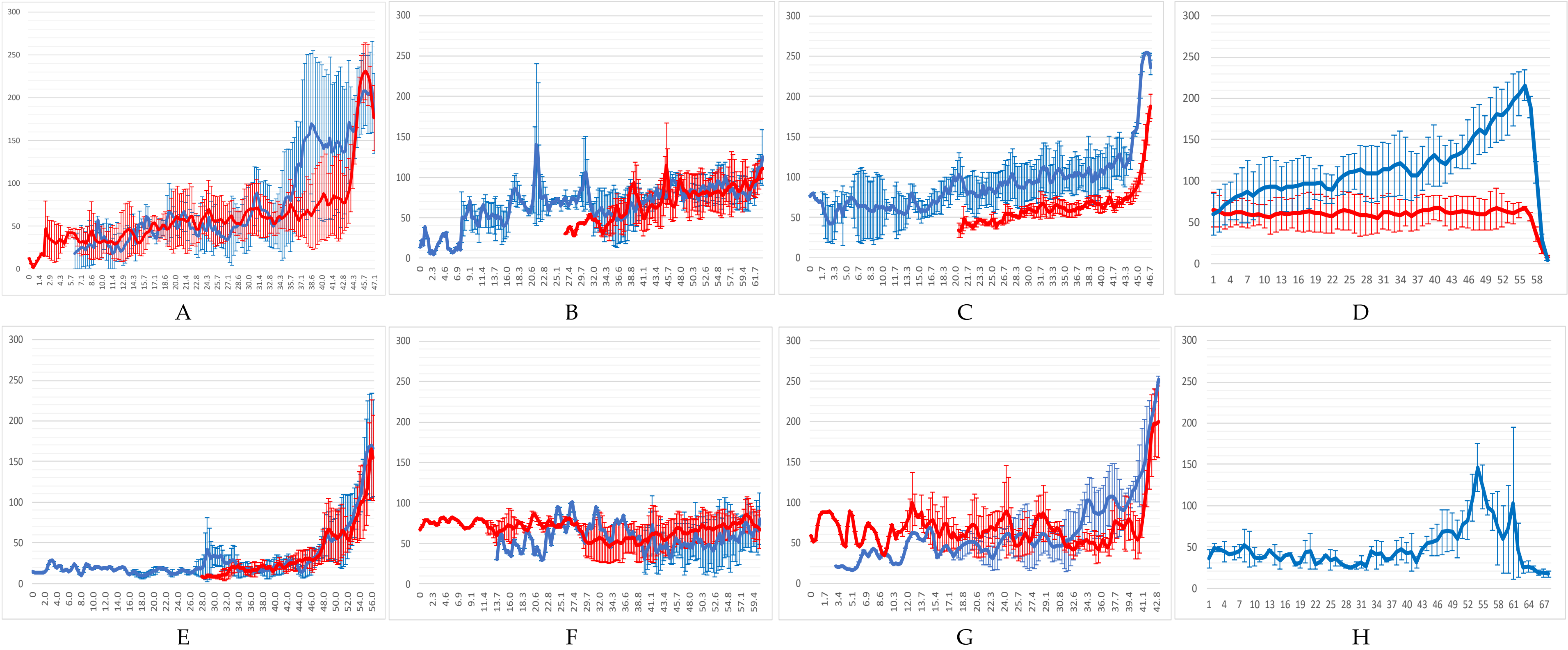

Supplemental Figure S1. Quantification of the intensity of the fluorescence (y-axis) of the GmFWL1-GFP (**A–D**, **H**) and mCherry-GmFLOT2/4 proteins (**E, F** and **G**) in mock- (Red) and *B. diazoefficiens*-inoculated (Blue) soybean root hair cells (x-axis, μm), under treatments of Auxin (**A** and **E**), Cytokinin (**B** and **F**), SA (**C** and **G**), and in response to L-kynurenine (**D**) or L-kynurenine and 2,3,5-triiodobenzoic acid (**H**). The figure showed average intensity of 3 root hair cells, and the error bar showing the standard error of the intensity of the fluorescence of the GFP and mCherry proteins.
